# Dietary Effects on Biological Parameters and Gut Microbiota of *Harmonia axyridis*

**DOI:** 10.3389/fmicb.2021.818787

**Published:** 2022-01-27

**Authors:** Zhendong Huang, Li Zhu, Jia Lv, Zhanxu Pu, Lipin Zhang, Guoqing Chen, Xiurong Hu, Zhenyu Zhang, Hongyu Zhang

**Affiliations:** ^1^The Citrus Research Institute of Zhejiang Province, Taizhou, China; ^2^State Key Laboratory of Agricultural Microbiology, Institute of Urban and Horticultural Entomology, College of Plant Science and Technology, Huazhong Agricultural University, Wuhan, China

**Keywords:** *Harmonia axyridis*, gut microbiota, biological parameters, food sources, diversity, influence

## Abstract

The multicolored Asian lady beetle (*Harmonia axyridis*, *H. axyridis*, Coleoptera, and Coccinellidae) is an effective biocontrol agent against agricultural pests. Previous studies have suggested that amount, type, and the quality of food can directly affect the biological aspects of *H. axyridis.* In this study, we investigated the influence of the food sources (*Acyrthosiphon pisum* Harris, *Diaphorina citri* Kuwayama, and artificial diets) on the gut microbiota diversity and the biology, reproductive variables, and population growth indicators of *H. axyridis.* Three kinds of diets were considered in this study: (1) HY: the adult of *A. pisum* Harris (HY group); (2) HM: the adult of *D. citri* Kuwayama (HM group); (3) HR: artificial diets prepared by blending a portion of fresh homogenized pork liver (15 g), honey (3 g), distilled water (35 ml) (HR group). We found that gut microbiota composition and diversity and the biological parameters differed when *H. axyridis* was fed with different diets. The abundance of *Enterobacteriaceae* was the highest in the HM group, followed by HY group, and was the lowest in the HR group. The abundance of *Staphylococcaceae* was highest in the HR group. Among the gut fungi, *Davidiellaceae* and *Wallemiaceae* were the highest and lowest in the HY group; *Incertae_sedis* were the major gut fungi in the HR group. Meanwhile, the changes of biological parameters may be correlated with the changes of *Streptococcaceae* abundance, *Micrococcaceae* abundance, *Staphylococcaceae* abundance, and *Enterobacteriaceae* abundance in responds to diet changes. To sum up, these data suggest that different diets can influence the changes in adult *H. axyridis* gut microbiota, consequently affecting the biological parameters.

## Introduction

The multicolored Asian ladybeetle, *Harmonia axyridis* (Pallas) (*H.axyridis*, Coleoptera: Coccinellidae), is an invasive generalist predator native to Southeast Asia, which has been used as an effective biocontrol agent against agricultural pests (Huang et al., 2019; Ricupero et al., 2020). *H. axyridis* can consume over 77 different prey species (found in approximately 85 plant species in 35 families). Their prey includes coccids, aphids, psyllids, and they even consume pollen and fruits (Koch, 2003; Soares et al., 2004; Koch et al., 2006; Martins et al., 2009; Guedes and Almeida, 2013). These aliments increase energy and assist in migration, providing survival in periods of prey scarcity and improving reproductive capacity. However, the amount and the quality of food are very important since they directly affect the biological aspects of *H. axyridis* (Evans and Dixon, 2000). When the ingested food is low or of poor quality, the development time usually increases, and the reproductive rates, i.e., oviposition, fecundity, and fertility, decrease (Sighinolfi et al., 2008; Castro-Guedes et al., 2016).

Polyphagous species have an imprinted role in integrated pest control because they can be more easily mass-produced with artificial diets, or even their numbers increase considerably in the field with alternative food sources (Hodek and Honek, 1996; Guedes and Almeida, 2013). Therefore, studying the effects of different diets on *H. axyridis* development and population maintenance is very important for mass production and using *H. axyridis* as a biocontrol agent.

Food has an important role in regulating the diverse compositions and structures of gut microbiota. The gut microbiota of insects has important roles during the life cycle of the host arthropod by promoting host growth and development, reproduction, speciation, immunity, and defense against predators (Oliver et al., 2014; Macke et al., 2017). Numerous studies have suggested that different diets affect the compositions and structures of the gut microbiome and, in turn, influence the host insect protective, metabolic, trophic, and immunological functions (Engel and Moran, 2013; Purchiaroni et al., 2013; Douglas, 2015; Ng et al., 2018; Omondi and Demir, 2021). However, so far, only a few studies have reported on gut microbiota in *H. axyridis* exposed to different diets (Dudek et al., 2017); most studies have been focused on the efficacy of bio-control strategies.

Pea aphid (*Acyrthosiphon pisum* Harris, *A. pisum*, Hemiptera: Aphididae) and Asian citrus psyllid (*Diaphorina citri* Kuwayama, *D. citri*, Hemiptera: Liviidae) are typical preys of *H. axyridis* and two of the most destructive pests of various crops like crops, vegetables, and fruits (Castro-Guedes et al., 2016). The sap-sucking *D. citri* is one of the most serious agricultural pests in the citrus-growing regions around the world. *D. citri* is the vector for *Candidatus Liberibacter asiaticus* (*CLas*), pathogenic bacteria that causes the highly destructive huanglongbing (HLB) disease, also known as citrus greening (Grafton-Cardwell et al., 2013; Meng et al., 2019). *H. axyridis* predators are considered the most important biological control agents of *D. citri*, which significantly contribute to its mortality in the field (Michaud and Olsen, 2004; Qureshi and Stansly, 2009; Huang et al., 2019). *A. pisum* is one of the key pests of pulse crops worldwide. The aphid has a broad host range, infesting crops such as faba bean, lupine, alfalfa, lentil, chickpea, grass pea, and pea (Sandhi and Reddy, 2020).

Over the years, a lot of research work has been done on the rearing technique of *H. axyridis* on artificial diets (Sighinolfi et al., 2008, 2013). Artificial diets were used as food, which furthered our knowledge of how artificial diets affect the gut microbiota, helping us develop the probiotics for artificial rearing *H. axyridis.*

In this study, we investigated the influence of the food sources (*A. pisum*, *D. citri*, and artificial diets) in shaping the gut microbiota diversity and the biology, reproductive variables, and population growth indicators of *H. axyridis.* We discovered that the core gut microbiota contributes to the development and populations maintenance of *H. axyridis*. This study furthers our knowledge on the co-adaptation relationship for insect adaptation to the food source and helps harness the *H. axyridis* symbiotic microbes for the artificial rearing and field population establishment and development of efficient development biocontrol strategies.

## Materials and Methods

### Experimental Design and Diets

The *H. axyridis* was obtained by the Huazhong Agricultural University (HZAU) in Hubei Province, China. Three kinds of diets were considered in this study: (1) HY: the adult of *A. pisum* (HY group); (2) HM: the adult of *D. citri* (HM group); (3) HR: artificial diets prepared by blending a portion of homogenized fresh pork liver (15 g), honey (3 g), distilled water (35 ml) (HR group).

Egg clusters of the *H. axyridis* from conventional rearing were collected, and newly hatched larvae were individually placed in a 55 mm × 15 mm plastic Petri dish to avoid cannibalism. Other treatments were tested in a 90 mm × 15 mm plastic Petri dish. *A. pisum* was reared on *Pisum sativum* L. seedlings in the laboratory and maintained at 25 ± 1°C, 70 ± 5% relative humidity (RH), and a photoperiod of 16L:8D under soft white fluorescent light. *D. citri* were collected from a citrus orchard in the Zhejiang province Huangyan district (28°38′38″N and 121°09′34″E) and were reared on *Murraya paniculata* L. Jack in the glasshouse at 25 ± 5°C with 50–70% RH and a photoperiod of 16L:8D dark. After the *H. axyridis* eggs hatched, the larvae were individually placed in Petri dishes lined with filter paper to begin the experiments. The food were replaced daily using sterile instruments. Small *A. pisum* and *D. citri* eggs were offered for the 1st instar larvae, medium-size and adult *A. pisum*, and *D. citri* for the other instars. After adult *H. axyridis* emergence, they were maintained in couples until the end of the experiments. The exchange and cleaning of the containers were done every 48 h and the observations were performed daily. All insects tested here were reared in laboratory conditions with daylight fluorescent neon tubes (Philips 30 W/33). The light intensity in close proximity of the testing arena was approximately 1,000 lx.

### Biological Parameters Measurement

Three different kinds of diets were used for rearing *H. axyridis* throughout the preimaginal development from incubation. Subsequently, one male and one female adult were paired. The following parameters were evaluated: (1) development time (days) from the first instar to adult emergence; (2) adult longevity (days); (3) pre-oviposition duration (days); (4) the percentage of the first instar larvae hatched from eggs; (5) total number of eggs/female laid, and a number of eggs/female laid per day; (6) oviposition duration (days); (7) survival probability of larva. Only the couples, including females who oviposited at least one fertile egg, were considered in the final result evaluation. For each treatment, 40 larvae (preimaginal parameters) or 10 couples (adult parameters) were considered as a replicate used for biological parameters measurement. There were 3 replicates for each diet.

### Gut Sample Collection

The whole gut from the adult of *H. axyridis* exposed to different diets was dissected. Briefly, the adult surface was sterilized by immersion in 70% ethanol for 3 min and rinsed three times in sterile phosphate-buffered saline. Four independent cohorts of adults were then dissected and used as biological replicates. Tissue samples were homogenized in an automatic sample Precellys-24 homogenizer (Shanghai Jingxin Industrial Development Co., Ltd., Shanghai, China) at 70 Hz/s for 60 s with a 10 s interval. Ten adult *H. axyridis* gut were dissected as a replicate; there were 3 replicates for each diet.

### DNA Extraction

DNA was extracted from adult *H. axyridis* gut using MagPureStool KFkit B (Magen, China) following the manufacturer’s instructions. Then quantified with a Qubit dsDNA BR Assay kit (Invitrogen, United States), and the quality was checked by running an aliquot on 1% agarose gel.

### Library Construction

Variable regions V4 of bacterial 16S rRNA gene were amplified with degenerate PCR primers: 515F (5′-GTGCCAGCMGCCGCGGTAA-3′) and 806R (5′-GGACTACHVGGGTWTCTAAT-3′). Both forward and reverse primers were tagged with illumine adapter, pad, and linker sequences. PCR enrichment was performed in a 50 μL reaction containing a 30 ng template, fusion PCR primer, and PCR master mix. PCR cycling conditions were: 95°C for 3 min, 30 cycles of 95°C for 45 s, 56°C for 45 s, 72°C for 45 s, and final extension for 10 min at 72°C for 10 min. The PCR products were purified using Agencourt AMpure XP beads and eluted in an Elution buffer. Libraries were qualified by the Agilent Technologies 2100 bioanalyzer. The validated libraries were used for sequencing on illumine Hiseq 2500 platform (BGI, Shenzhen, China) following the standard pipelines of illumine, generating 2 × 250 bp paired-end reads.

The ITS1 of Internal Transcribed Spacer (ITS) region was amplified with degenerate PCR primers, ITS1 (5′-CTTGGTCATTTAGAGGAAGTAA-3′) and ITS2 (5′-GCTGCGTTCTTCATCGATGC-3′). Both primers were tagged with illumine adapter, pad, and linker sequences. PCR enrichment was performed in a 50 μL reaction containing a 30 ng template, fusion PCR primer, and PCR master mix. PCR cycling conditions were: 94°C for 3 min, 30 cycles of 94°C for 30 s, 55°C for 45 s, 72°C for 45 s, and final extension for 10 min at 72°C for 10 min. The PCR products were purified with AMpureXP beads and eluted in an Elution buffer. Libraries were qualified by the Agilent 2100 bioanalyzer (Agilent, United States). The validated libraries were used for sequencing on illumine Hiseq 2500 platform (BGI, Shenzhen, China) following the standard pipelines of illumine, generating 2 × 250 bp paired-end reads.

### Data Processing and Bioinformatics

Raw sequences were deduplicated and quality-filtered by Fastp version 0.20.0 (Chen et al., 2018). After filtering low-quality sequences according to previous criteria (Gill et al., 2006), paired-end reads were merged using Flash version1.2.11 (Magoc and Salzberg, 2011). After chimera detection, all remaining high-quality sequences were clustered into operational taxonomic units (OTUs) with a similarity threshold of 97% sequence identity by Usearch version 7.0.1090 (Edgar, 2013). The Greengenes Database (DeSantis et al., 2006) and the Unite Database (Kõljalg et al., 2013) were used for BLAST searching with the representative sequences and further taxonomy classification. The rarefaction analysis based on Mothur software Version 1.21.1 was conducted to reveal the diversity indices, including Chao, ACE, Shannon and Simpson diversity index diversity indices (Schloss et al., 2009). The coverage of the predicted diversity in each clone library was calculated using the formula *C* = [1 − (*n*_1_/*N*)] × 100%, where *N* is the total number of clones, and *n*_1_ is number of OTUs appearing only once in the library (Good, 1953). The species accumulation curves using the R package version 3.2.1, the beta diversity analysis was performed with UniFrac to compare the results of PCA using the R package version 3.1.1 (Oksanen et al., 2010). Venn diagrams were implemented using the Venn Diagram R package. Mantel test, RDA, and heatmap generation were performed in Vegan packages in UPGMA mean clustering using the R package version 3.1.1 “VennDiagram” (R Core Team, 2002). We used the Galaxy implementation of LEfSe^1^ with Clustergram. Phylogenetic investigation of communities by reconstruction of unobserved states (PICRUSt) was performed to predict microbial functions using the high-quality sequences using the R package version 3.4.1 (Langille et al., 2013). Using the Spearman algorithm in the psych package to calculate the correlation coefficient about the relationship between biological parameters and gut microbiota, then construct heat map used by pheatmap^2^.

### Statistical Analysis

**FIGURE 1:**
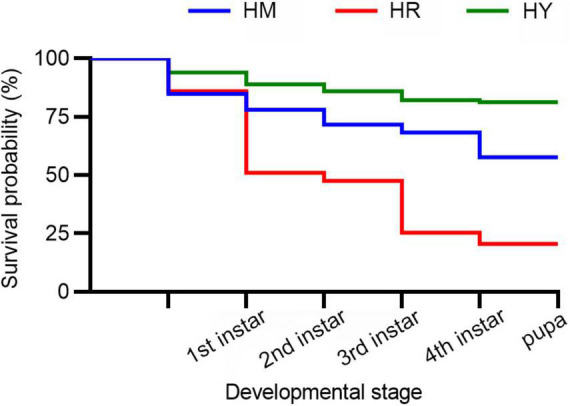
The survival probability of *H. axyridis* across the life stages fed with three different diets.

One-way analysis of variance by Tukey’s test was used to calculate the statistical significance among different groups, The resultant data from each experiment were analyzed using SPSS version 20.0 software with advanced models (SPSS Japan Inc., Tokyo, Japan). The mean values of replicates were expressed as mean ± standard error (SE, *n* = 3), and *P*-value < 0.05 was considered statistically significant. Excel 2010 software and GraphPad Prism 8.0 (GraphPad Software, La Jolla, CA, United States) were used to generate Figures 1, 6.

## Results

### Development Period

The mean incubation period was shorter in the HY group compared to the HM group (2.88 ± 0.083 vs. 4.81 ± 0.072; *F* = 411.857; *P* < 0.01) (Table 1). In addition, there was a difference in the development period in the 3rd and 4th instar among the three dietary treatments; HY group had the shortest time (3rd: *F* = 84.912; *P* < 0.01; 4th: *F* = 42.213; *P* < 0.01). For individuals fed with HY diet, the pupa stage was shorter (5.63 days) than in HM (6.15 days) and significantly shorter HR groups (6.50 days; *F* = 17.256; *P* = 0.025). Also, the total development period from 1st instar to adult emergence was 19.78 days in the HY group compared to 22.39 and 23.42 in the HM and HR group, respectively, and significantly shorter than in HR (*F* = 9.025; *P* = 0.016) (Table 1).

**TABLE 1:** Development period in days (mean ± the standard error of the mean) of *H. axyridis* (Pallas, 1773) fed with three different diets: (1) HY, *A. pisum*; (2) HM, *D. citri*; (3) HR, artificial diets; at 25 ± 1°C, 70 ± 5% RH and a photoperiod of 16L:8D.

Development period	Diets		
	HY	HM	HR
Incubation period	2.88 ± 0.083 a	4.81 ± 0.072 b	–
1st instar	2.48 ± 0.051	2.66 ± 0.200	2.92 ± 0.306
2nd instar	3.64 ± 0.323	3.74 ± 0.225	3.57 ± 0.277
3rd instar	2.89 ± 0.067 a	4.15 ± 0.064 c	3.67 ± 0.078 b
4th instar	4.40 ± 0.213 a	5.68 ± 0.199 b	6.77 ± 0.168 c
Pupa	5.63 ± 0.267 a	6.15 ± 0.191ab	6.50 ± 0.115 b
Total	19.78 a	22.39 ab	23.42 b

*The values were shown as mean ± standard error (SE), the different small letter in rows after the value showing the significant level using Tukey’s test (P < 0.05).

### Longevity and Reproductive Parameters

The longevity of adult *H. axyridis* was similar when using HY and HM dietary treatments (*F* = 1.267; *P* = 0.3773) but shorter when HR dietary treatment was applied (*F* = 13.284; *P* = 0.0171) (Table 2). The pre-oviposition period was shorter (5.17 days vs. 14.92 days; *F* = 232.783; *P* = 0.0006) and the hatch ratio (58.89 ± 1.862 vs. 77.14 ± 3.418; *F* = 25.311; *P* = 0.0151) was smaller in the HY group compared to the HM group. In addition, the mean number of eggs produced per female (279.80 ± 4.950 vs. 193.27 ± 1.633; *F* = 650.189; *P* = 0.0000) and mean daily number of eggs produced per female (9.71 ± 0.226 vs. 6.21 ± 0.127; *F* = 4.128; *P* = 0.1373) was higher in the HY group vs. HM group, while there was no difference in the oviposition period (Table 2).

**TABLE 2:** Reproductive parameters and longevity (days) (mean ± standard error of the mean) of *H. axyridis* (Pallas, 1773) feeding with three different diets: (1) HY; (2) HM; and (3) HR; at 25 ± 1°C, 70 ± 5% RH, and a photoperiod of 16L:8D.

Variables	Diets		
	HY	HM	HR
Longevity (days)	34.63 ± 0.497 a	37.50 ± 3.590 a	27.23 ± 0.549 b
Pre-oviposition (days)	5.07 ± 0.082 a	14.93 ± 0.082 b	–
Hatch ratio/%	58.89 ± 1.862 a	77.14 ± 3.418 b	–
Eggs numbers/per female	279.80 ± 4.950 a	193.27 ± 1.633 b	–
Eggs/day	9.71 ± 0.226 a	6.21 ± 0.127 b	–
Oviposition duration (days)	28.60 ± 0.245	31.60 ± 2.902	–

*The values were shown as mean ± standard error (SE), the different small letter in rows after the value showing the significant level using Tukey’s test (P < 0.05).*

### Survival Probability of Larva on Different Diets

In HY group, the survival probability of 1st instar to 4th instar larva and pupa were higher than in the HM and HR groups (*p* < 0.05) (Figure 1).

### Sequencing Summary

**FIGURE 2:**
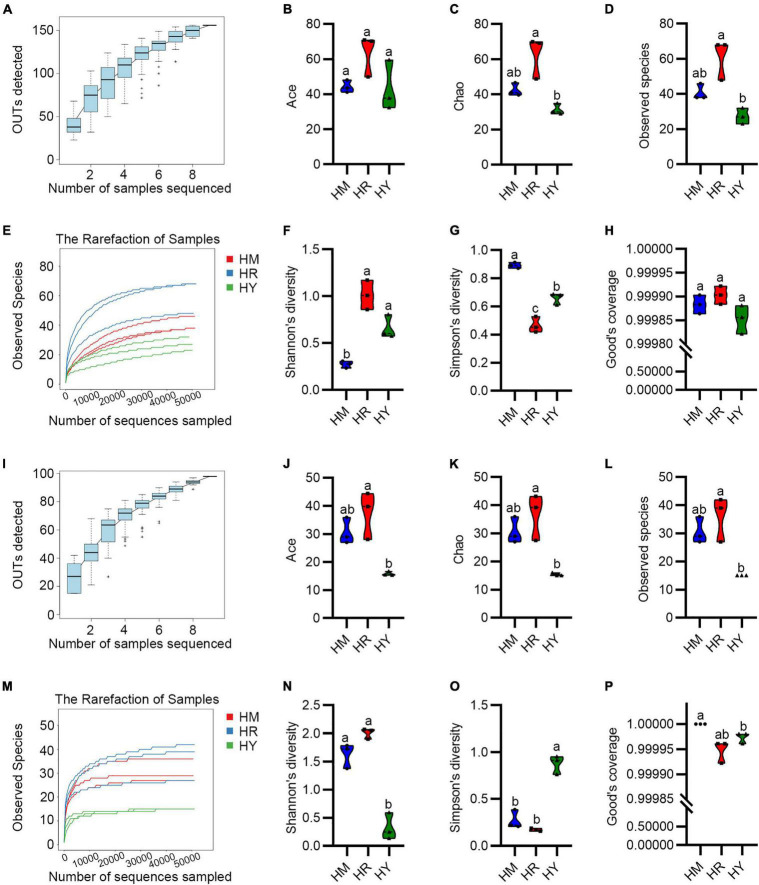
The Alpha diversity of intestinal microbiota in adult *H. axyridis* fed with different diets. **(A–H)** The Alpha diversity of bacteria. **(I–P)** The Alpha diversity of fungi. The different small letter above bars indicate significant difference by Tukey’s test (*P* < 0.05).

The sequencing data yielded a total of 457,730 reads of bacterial 16S rRNA genes and 458,913 reads of the ITS region of fungal rRNA genes with average lengths of 293 and 297bp (Supplementary Tables 1, 2), respectively. The tendencies of the species accumulation curves tended toward saturation (Figures 2A,I), which implied that the samples could reveal the microbial communities. The rarefaction curve of the bacterial (Figure 2E) and fungal (Figure 2M) leveled off after the total number of sequences reached 15,000 in the sequencing process, after which they became smooth, indicating that the sequencing amount was more reasonable.

### Diversity and Richness Estimates

All the Good’s coverage index tended to be 1 (Figures 2H,P), which suggested that all the samples were adequately sequenced. Ace (Figures 2B,J), Chao (Figures 2C,K), and observed OTUs (Figures 2D,L) showed that the bacterial and fungal community diversity of the HM and HR groups were higher than that of HY group, but without significant difference.

Shannon’s diversity and Simpson’s diversity were used to evaluate the species diversity. The bacterial Shannon’s diversity of the HM group was significant lower compared with the HR and HY group (Figure 2F), while the fungal Shannon’s diversity of the HY group was significant lower compared with the HM and HR group (Figure 2N), whereas bacterial and fungal Shannon’s diversity of the HR group was higher than in the HY group. Moreover, Simpson’s diversity analysis showed the bacterial Simpson’s diversity of the HR group was significant lower compared with the HM and HY group, meanwhile, HM group was significant higher compared with the HR and HY group (Figure 2G), while the fungal Simpson’s diversity of the HY group was significant higher compared with the HM and HR group (Figure 2O). Similarly, the bacterial and fungal Simpson’s diversity of the HY group was also significant higher than that in the HR group.

### The Composition of the Gut Microbiota

**FIGURE 3:**
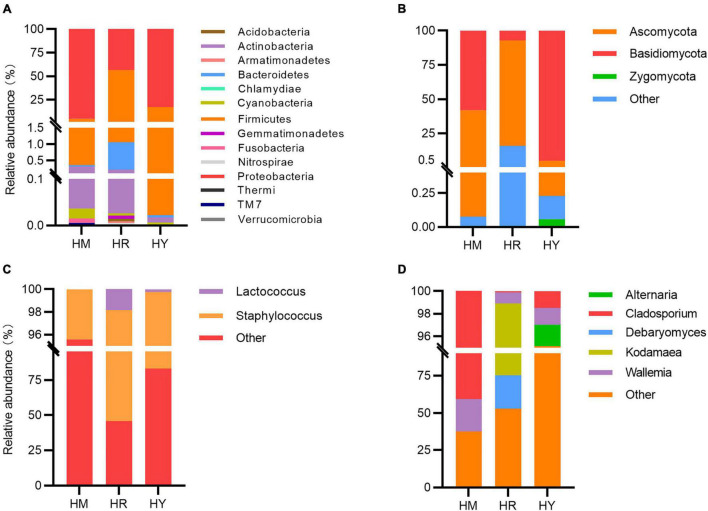
Relative abundance of the most abundant taxa at the family or genus levels in the guts of *H. axyridis* fed with different diets. Bacterial taxonomic compositions at the phylum level are shown in panel **(A)** and at the genus level **(C)**; fungi taxonomic compositions at the phylum level are shown in panel **(B)** and at the genus level **(D)**.

The gut bacteria of adult *H. axyridis* fed with different diets were mainly affiliated with 14 phyla. In the HM group, *Proteobacteria* (95.19% of reads) was the most common bacteria, followed by *Firmicutes* (4.45% of reads), *Actinobacteria* (0.0028%), *Bacteroidetes* (0.00043%), and *Cyanobacteria* (0.00022); in the HY dietary group, *Proteobacteria* (82.92% of reads) was the most common bacteria, followed by *Firmicutes* (17.05% of reads), *Actinobacteria*, *Bacteroidetes*, *Cyanobacteria*, and *Thermi*; in the HR group, the two dominant phyla were *Firmicutes* (55.30%) and *Proteobacteria* (43.64%), followed by *Bacteroidetes* (0.0084%), and *Actinobacteria*, *Cyanobacteria*, *Gemmatimonadetes*, *Acidobacteria*, *Armatimonadetes*, *Nitrospirae*, *Chlamydiae*, and *Verrucomicrobia* (Figure 3A).

We further investigated the fungal composition. Taxonomic analysis at the phylum level revealed that compositions of fungi were different in the three groups. *Basidiomycota* and *Ascomycota* were found in all groups. *Basidiomycota* (95.01%) was the most prevalent phylum in the HY group, while the abundance of *Ascomycota* (76.90%) was highest in HR dietary treatments. In the HM group, the relative abundances of *Basidiomycota* and *Ascomycota* accounted for 58.09 and 41.83%, respectively (Figure 3B).

The abundance of *Proteobacteria* was highest in the HM group, followed by the HY group, and was the lowest in the HR group. The abundance of *Firmicutes* was highest in the HR group, followed by the HY group, and the lowest in the HM group. The abundance of *Basidiomycota* was highest in the HY group, followed by HM group, and the lowest in the HR group. The abundance of *Ascomycota* was highest in the HR group, followed by the HM group, and was the lowest in the HY group.

At the genus level, In the HY group, *Staphylococcus* (16.69%) and *Lactococcus* (0.25%) showed high abundance; in the HR group, the content of *Staphylococcus* (52.57%) was highest, followed by *Lactococcus* (1.82%); In the HM group, the content of *Staphylococcus* (4.42%) was highest (1.82%) (Figure 3C). Among fungi in the gut, the highest abundance of *Cladosporium* (40.64%) and *Wallemia* (21.86%) was found in HM group, while *Cladosporium* (1.47%) and *Wallemia* (1.51%) were sharply decreased in the HY group. In the HR group, *Kodamaea* (23.64%) and *Debaryomyces* (22.41%) were the major gut fungi (Figure 3D).

**FIGURE 4:**
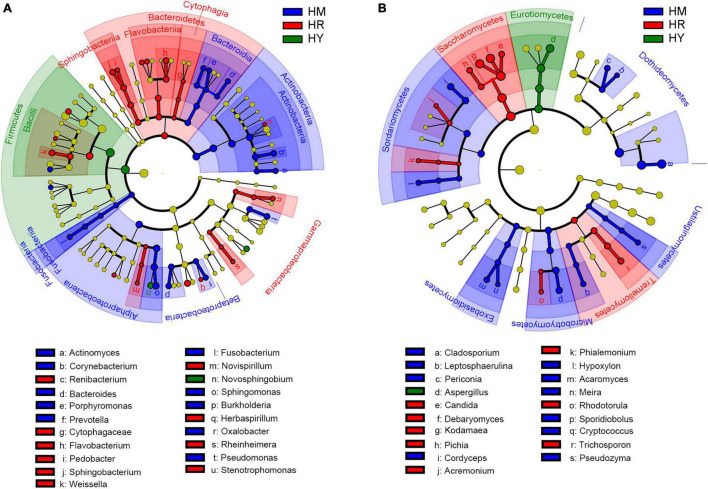
LEfSe analysis of intestinal microbiota in *H. axyridis* fed with different diet. **(A)** LEfSe analysis of bacteria. **(B)** LEfSe analysis of fungi.

LEfSe analysis, namely LDA Effect Size analysis, can realize the comparison between multiple groups, and conduct subgroup comparison analysis within group comparison, so as to find the species with significant differences in abundance between groups. In the HM group, *Actinomyces, Corynebacterium, Bacteroides, Porphyromonas, Prevotella, Fusobacterium, Sphingomonas, Burkholderia, Oxalobacter*, and *Pseudomonas* were the main bacteria, and such as *Corynebacterium, Sphingomonas, Pseudomonas* likely played important roles. *Renibacterium, Cytophagaceae, Flavobacterium, Pedobacter, Sphingobacterium, Weissella, Novispirillum, Herbaspirillum, Rheinheimera*, and *Stenotrophomonas* were the main bacteria in the HR group. *Flavobacterium, Sphingobacterium, Weissella* likely had important roles in HR group. *Novosphingobium* likely played an important role in the HY group (Figure 4A).

*Cladosporium, Leptosphaerulina, Periconia, Cordyceps, Hypoxylon, Acaromyces, Meira, Sporidiobolus, Cryptococcus*, and *Pseudozyma* were the major fungal group in the HM group, and fungus such as *Cladosporium, Cordyceps*, and *Acaromyces* may have important roles. *Aspergillus* was the major groups in the HY group. *Candida, Debaryomyces, Kodamaea, Pichia, Acremonium, Phialemonium, Rhodotorula*, and *Trichosporon* were the major fungal group in the HR group; some fungi such as *Debaryomyces, Kodamaea* and *Trichosporon* likely have important roles (Figure 4B).

### Correlation of Gut Microbiota in *H. axyridis* Fed With Different Diet

**FIGURE 5:**
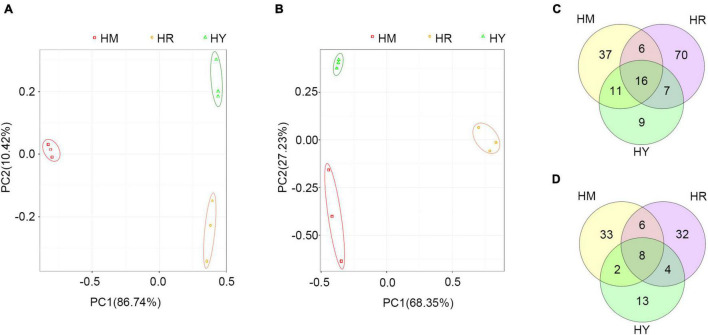
The PCA analyses and Venn diagram analysis in the intestine of adult *H. axyridis* fed with different diets. **(A,B)** PCA analyses of bacteria **(A)** and fungus **(B)** at OUT. **(C,D)** The overlap of bacterial OTU **(C)** and fungal OTU **(D)**.

The PCA plot of bacterial communities (Figure 5A), based on the relative abundance of OTU in different treatments, showed variation in the profiles of the first component (PC1) (86.74%) and a second component (PC2) (10.42%). While fungal communities (Figure 5B) showed variation in the profiles of the first component (PC1) (68.35%) and a second component (PC2) (27.23%). These results suggested that different dietary treatments led to differences in the composition of gut microbiota among treatments.

**FIGURE 6:**
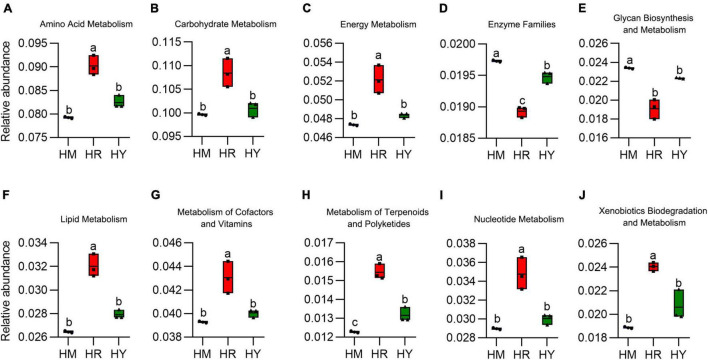
**(A–J)** The relative abundance of intestinal microbiota metabolism from adult *H. axyridis* fed with different diets. The different small letter above bars indicate significant difference by Tukey’s test (*P* < 0.05).

Venn diagram analysis was used to obtain the overlap of OTUs in different treatments. The results showed that HM and HY groups shared 27 bacterial OTUs and 10 fungal OTUs, respectively (Figures 5A,B). Most of the shared bacterial OTUs belonged to *Enterobacteriaceae*, *Staphylococcaceae*, and *Pseudomonadaceae*, while most of the fungal OTUs belonged to *Wallemiaceae*, *Davidiellaceae*, and *Trichocomaceae* (Supplementary Table 3). The HR and HY groups shared 23 bacterial OTUs and 12 fungal OTUs (Figures 5C,D); most of the shared bacterial OTUs belonged to *Enterobacteriaceae*, *Staphylococcaceae*, *Moraxellaceae*, and *Pseudomonadaceae*, while most of fungal OTUs belonged to *Wallemiaceae*, *Davidiellaceae*, *Pleosporaceae*, and *Trichocomaceae* (Supplementary Table 4). The HR and HM groups shared 22 bacterial OTUs and 14 fungal OTUs (Figures 5C,D); most of the shared bacterial OTUs belonged to *Enterobacteriaceae*, *Staphylococcaceae*, *Moraxellaceae*, and *Pseudomonadaceae*, while most of shared fungal OTUs belonged to *Davidiellaceae*, *Trichocomaceae* (Supplementary Table 5).

### Phylogenetic Investigation of Communities by Reconstruction of Unobserved States Metagenomic Predictions

The HR diet significantly promoted relative abundance of amino acid, carbohydrate, energy, nucleotide, lipid, metabolism of cofactors and vitamins metabolism, reduced the glycan biosynthesis and enzymes. There was no significant difference between the HM and HY diets, except that enzyme families were higher in the HM group vs. HY group (Figures 6A–J).

### Relationships Between the Changes in Abundance of Gut Microbiota and the Changes of Biological Parameters in *H. axyridis* Fed With Different Diet

**FIGURE 7:**
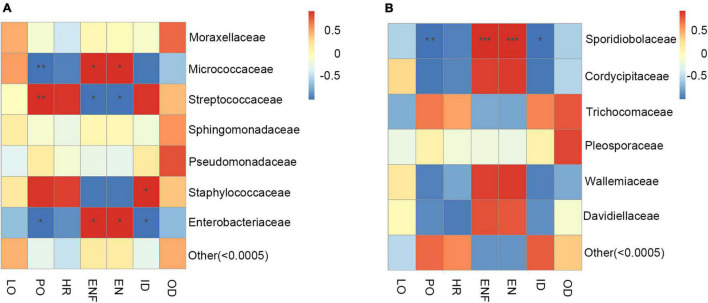
The correlation heatmap of gut microbiota and biological parameters. **(A)** Bacterial at the family level. **(B)** Fungus at the family level. Red represents positive correlation; blue represents negative correlation, * shows 0.01 < *p* < 0.05, ** shows 0.001 < *p* < 0.01, *** shows *p* < 0.001. LO, longevity; PO, pre-oviposition; HR, hatch ratio; ENF, eggs numbers/per female; EN, eggs/day; ID, incubation period; OD, oviposition duration.

In addition, the correlation heat map was used to explore the connection between the changes in the abundance of gut microbiota and the biological parameters of adult *H. axyridis*. As shown in Figure 7A, at the family level, the changes in abundance of *Micrococcaceae* were negatively correlated with changes in pre-oviposition duration (*P* = 0.0069), and positively correlated with changes in eggs numbers/per female (*P* = 0.0144) and eggs/day (*P* = 0.0133). Contrary, *Streptococcaceae* abundance was positively correlated with changes in pre-oviposition duration (*P* = 0.0020) and negatively correlated with changes in eggs numbers/per female (*P* = 0.0212) and eggs/day (*P* = 0.0301). The changes in abundance of *Staphylococcaceae* were positively correlated with changes in incubation period (*P* = 0.0114); changes in abundance of *Enterobacteriaceae* was negatively correlated with changes of pre-oviposition duration (*P* = 0.0422) and incubation period (*P* = 0.0422) and positively correlated with changes in eggs numbers/per female (*P* = 0.0235) and eggs/day (*P* = 0.0203).

For the connection between the changes in the abundance of gut fungal community and the biological parameters of adult *H. axyridis*, at the family level, the changes in abundance of *Sporidiobolaceae* was significantly negatively correlated with changes of pre-oviposition duration (*P* = 0.0073) and incubation period (*P* = 0.0310) and positively correlated with changes in eggs numbers/per female (*P* = 0.0002) and eggs/day (*P* = 0.0005) (Figure 7B).

## Discussion

Food supply is considered essential for the complete development and reproduction of *H. axyridis*. Differential diets with varied nutritional constituents influence insect development time, reproduction, and longevity (Evans et al., 1999; Clercq et al., 2005; Jalali et al., 2009). Our experiment suggested that *H. axyridis* could complete its development and reproduction when fed with *A. pisum* and *D. citri*, which was not possible when using an artificial diet prepared by blending a portion of fresh pork liver, honey, and distilled water. This may be because the diet lacks vitamin mixture and the water amino acid solution (Sighinolfi et al., 2008). Recently, Intazar et al. (2016) also found that emerged ladybird adults did not lay eggs when fed with artificial diets; however, oviposition was restored when adults were given *A. pisum* (Intazar et al., 2016). Moreover, in a previous study, *H. axyridis* was reared on liver-based artificial diets or *Ephestia kuehniella* eggs, after which the survival rates decreased, and larval development time increased (Sighinolfi et al., 2008, 2013).

Castro-Guedes et al. (2016) found that the type of food influences the development, bodyweight, longevity, and reproductive variables of *H. axyridis*. In this study, we found that the mean incubation period was shorter in the *A. pisum* group compared to the *D. citri* group. In addition, there was a difference in the development period in the 3rd and 4th instar among the three dietary treatments; *A. pisum* group had the shortest time. Moreover, for individuals fed with *A. pisum* diet, the pupa stage was significantly shorter than in *D. citri* and *A. pisum* groups. It was showed that the microbial composition, nutrient content of *A. pisum* was more suitable for the development of *H. axyridis.* Meanwhile, the period of pre-oviposition and hatch ratio were significantly shorter in the *A. pisum* group compared with the *D. citri* group, while the survival probability of 1st instar to 4th instar larva and pupa were higher. Simultaneously, the highest fertility was observed in individuals fed with *A. pisum*, thus demonstrating that the production of offspring per female during the lifetime of *H. axyridis* was optimized. *H. axyridis* usually consumes the thorax and abdominal parts of adult *D. citri*, leaving the head or wings. Aphids such as *A. pisum* are more easily handled and consumed by the predator and may be more suitable prey in terms of net energy gain (Huang et al., 2019). However, the potential impact of the microbiota on intestinal absorption of dietary remains unclear, although the microbiota can influence dietary nutrient harvest, the diet can also impact gut microbiota composition and function. Gut microbial community membership is correlated with diet composition in other vertebrates (Wu et al., 2011; Semova et al., 2012). In addition, we speculated the *A.pisum* as diet was adapted to be easily absorbed by gut microbiota of *H. axyridis*.

Over recent years, the coevolutionary relationship between hosts and their gut microbiota has become a research hotspot. Gut microbiota has an important role in energy budget (Semova et al., 2012), nutrient metabolism (Cani, 2016; Greer et al., 2016), immune homeostasis (Dimitriu et al., 2013), foraging behavior (Heijtz et al., 2011), and reproductive performance (Leftwich et al., 2017; Zhou et al., 2020). In this study, we analyzed the gut microbiota of *H. axyridis* fed with different diets. Venn diagram and LEfSe analysis indicated significant differences in species richness among different groups. PCA analysis also suggested significant differences in gut microbial community structure. These findings indicated that food might alter gut microbial structure and abundance in adult *H. axyridis*. Similarly, other studies have suggested that different diets may lead to changes in the gut microbiota composition of other insects (Dewar et al., 2014; Li et al., 2014; Wang et al., 2016; Mohammed et al., 2018). *Proteobacteria* was the most dominant bacteria in the HY and HM groups; *Firmicutes* was the most dominant phylum in *H. axyridis* fed with an artificial diet; these findings are similar to a previous study (Dudek et al., 2017). *Ascomycota* and *Basidiomycota* were found to constitute the main members of the fungal community.

*Proteobacteria* are associated with diverse metabolism and typical decomposition, fermentation of complex sugars, and vitamin production. They degrade a variety of aromatic compounds and boost the nutrient absorption of their hosts (Reid et al., 2011; Colston and Jackson, 2016). *Firmicutes* can encode the energy metabolism-related enzymes, potentially biosynthesize vitamin B, produce diverse kinds of digestive enzymes to break down various substances, helping their hosts to digest and absorb nutrients (Flint et al., 2012). The microbiota of the insect gut is influenced by several factors, such as the environmental habitat, diet, immune system, physiology, and phylogeny of the host (Yun et al., 2014; Yao et al., 2016; Martinson et al., 2017; Mistry et al., 2017). Considering that biological parameters of *H. axyridis* populations were shifted with different diets, it is highly likely that the diet had a role in shaping the gut microbial community structure of *H. axyridis*. Additionally, in this study, the abundance and composition of adults *H. axyridis* gut microbiota were affected by different diets. Our study showed that, despite a certain overlap, the bacterial and fungal communities in adults *H. axyridis* gut had distinct OTUs differences, most likely due to transient microbes acquired from diets but potentially also due to different stable microbial associations in the coevolutionary relationship between hosts and food.

The microbial communities within the adults *H. axyridis* perform many key functions, including amino acid transport and metabolism, carbohydrate transport and metabolism, transcription, and inorganic ion transport and metabolism, which are essential for the survival of insects (Liu et al., 2020). Our results demonstrated that gut microbiota are also important in adults *H. axyridis* development (Omondi and Demir, 2021), the artificial dietary treatment significantly promoted the relative abundance of amino acid, carbohydrate, energy, nucleotide, lipid, metabolism of cofactors, and vitamins metabolism, and reduced the relative abundance of glycan biosynthesis and enzyme families; however, there was no difference in *D. citri* and *A. pisum* dietary treatments, except that *D. citri* significantly increased the relative abundance of enzyme families compared to the *A. pisum* group. Bacterial cells may provide essential nutrients, such as vitamins and amino acids, and enzymes that digest indigestible components of the artificial diet and *D. citri* to more digestible and absorbable levels (Peterkova-Koci et al., 2012), the bacterial genomes were closely correlated with their natural environments, especially for the host diets. Thus, it may be suggested that food kinds have a more profound effect on the functional pathways of gut microbes in adult *H. axyridis*. The differences in the function prediction could be explained by the sugar, amino acid content, and secondary metabolites in the food resources of *H. axyridis*.

Strikingly, the correlation heat map was used to study the connection between the changes of gut microbiota abundance and the biological parameters of adult *H. axyridis*. *Micrococcaceae* abundance was negatively correlated with changes of pre-oviposition duration and positively correlated with changes of eggs numbers/per female and Eggs/day. *Micrococcaceae* have previously been identified as cellulolytic species. It has an important role in the digestion of cell walls and lignocelluloses into glycoside hydrolases (Shil et al., 2014; Hu et al., 2018). We speculate that *Micrococcaceae* may promote the absorption of nutrients during oviposition and mating of adult *H. axyridis*. Contrary, we found that *Streptococcaceae* abundance was positively correlated with changes of pre-oviposition duration, and negatively correlated with changes in eggs numbers/per female and eggs/day. *Staphylococcaceae* abundance was positively correlated with changes in incubation period; *Enterobacteriaceae* abundance was negatively correlated with changes of pre-oviposition duration and incubation period, and positively correlated with changes of eggs numbers/per female and eggs/day. In other words, *Enterobacteriaceae* could shorten the pre-oviposition duration and incubation period, and promote reproduction. *Enterobacteriaceae* are symbiotic microbes shared by human gut tracts and insect guts that often have important roles in vitamin biosynthesis, pheromone production, and degradation of plant compounds (Breznak, 1982; Xu and Gordon, 2003; Schloss et al., 2006). They are responsible for enzyme metabolism through superoxide dismutase or catalase enzyme activity (Xia et al., 2017), and this family has been reported to participate in sugar metabolism. Researchers have suspected that *Enterobacteriaceae* contributes to digestion, protection, courtship, and reproduction (Ami et al., 2010; Hu et al., 2018; Zhao et al., 2018).

Differences in the gut fungal community structure might be due to several selective factors, e.g., changes in behavior, microbial interactions, physicochemical and nutritional conditions (Adams et al., 2009; Morales-Jiménez et al., 2012; Briones-Roblero et al., 2017). The changes in abundance of *Sporidiobolaceae* were negatively correlated with changes in pre-oviposition duration and incubation period and positively correlated with changes in eggs numbers/per female and eggs/day in *H. axyridis* in this study. It is possible to hypothesize that *Sporidiobolaceae* is a key family that influence by the changes in behavior of *H. axyridis.*

## Conclusion

Our data suggest that different diets may affect the gut microbiota abundance, as well as the incubation period, development period, longevity, pupa stage, survival probability of 1st instar to 4th instar larva, and pupa and the period of pre-oviposition and hatch ratio in adult *H. axyridis*. The changes in biological parameters may be correlated with *Streptococcaceae* abundance, *Micrococcaceae* abundance, *Staphylococcaceae* abundance, and *Enterobacteriaceae* abundance. The gut microbiota of adult *H. axyridis* may be indispensable to their adaption to the foods and development of the host. Yet, more studies (e.g., metagenomic and identification approaches) are required to further analyze the interactions of hosts and microbiota, as well as microbial structure and function in *H. axyridis* fed with different diets. These findings further the understanding of the gut microbiota of this invasive generalist predator and may provide clues for the artificial rearing and field population establishment of *H. axyridis* and the development of efficient bio-control strategies.

## Data Availability Statement

The datasets presented in this study can be found in online repositories. The names of the repository/repositories and accession number(s) can be found in the article/Supplementary Material.

## Author Contributions

ZH and HZ contributed to the conceptualization of this study. JL, ZP, LPZ, GC, and XH performed the experiments. ZH and LZ assisted in the original draft preparation. ZH and LZ contributed to the manuscript—review and editing. ZH, LZ, and ZZ performed the analysis with constructive discussions. ZH and GC contributed to supervision, project administration, and funding acquisition. All authors have read and agreed to the published version of the manuscript.

## Conflict of Interest

The authors declare that the research was conducted in the absence of any commercial or financial relationships that could be construed as a potential conflict of interest.

## Publisher’s Note

All claims expressed in this article are solely those of the authors and do not necessarily represent those of their affiliated organizations, or those of the publisher, the editors and the reviewers. Any product that may be evaluated in this article, or claim that may be made by its manufacturer, is not guaranteed or endorsed by the publisher.

## Footnotes

1
https://huttenhower.sph.harvard.edu/galaxy/

2
https://cran.r-project.org/web/packages/psych/psych.pdf
