# Supplementary material for: Dietary Effects on Biological Parameters and Gut Microbiota of Harmonia axyridis
Source: Front Microbiol. 2022 Jan 27;12:818787. doi: 10.3389/fmicb.2021.818787 (PMC8828657; doi:10.3389/fmicb.2021.818787)
Supplement: Supplementary file 1 [file Table_1.DOCX]

**Table S1.** The OTU clustering results statistics of 16S rRNA

| Sample name | Tag number | OTU number |
| --- | --- | --- |
| HM1 | 51420 | 46 |
| HM2 | 51463 | 38 |
| HM3 | 51359 | 38 |
| HR1 | 50988 | 48 |
| HR2 | 51577 | 68 |
| HR3 | 51617 | 68 |
| HY1 | 48700 | 32 |
| HY2 | 50422 | 27 |
| HY3 | 50184 | 23 |

**Table S2.** The OTU clustering results statistics of ITS

| Sample name | Tag number | OTU number |
| --- | --- | --- |
| HM1 | 50941 | 36 |
| HM2 | 50949 | 27 |
| HM3 | 50854 | 29 |
| HR1 | 51041 | 39 |
| HR2 | 51037 | 42 |
| HR3 | 51074 | 27 |
| HY1 | 51026 | 15 |
| HY2 | 50946 | 15 |
| HY3 | 51045 | 15 |

**Table S3.** The common OTUs after supplying HM and HY to *H. axyridis*

| **Family** | **OTUs** | **Family** | **OTUs** |
| --- | --- | --- | --- |
| **Bacteria** |  |  |  |
| Enterobacteriaceae | OTU7; OTU1; | Staphylococcaceae | OTU2; OTU146;OTU9 |
| Pseudomonadaceae | OTU3;OTU134 | Micrococcaceae | OTU14 |
| Moraxellaceae | OTU12; OTU46 | Enterococcaceae | OTU13 |
| Sphingomonadaceae | OTU16;OTU15 | Bifidobacteriaceae | OTU24 |
| Burkholderiaceae | OTU31 | Corynebacteriaceae | OTU143;OTU114 |
| Rhodobacteraceae | OTU39 | Streptococcaceae | OTU82 |
| Propionibacteriaceae | OTU55 | Lactobacillaceae | OTU120 |
| Prevotellaceae | OTU115 | mitochondria | OTU144 |
| Bacteroidaceae | OTU151 | Tissierellaceae | OTU156 |
| undefined | OTU22; OTU44 |  |  |
| **Fungi** |  |  |  |
| Wallemiaceae | OTU2 | Davidiellaceae | OTU7; OTU26; OTU20 |
| Trichocomaceae | OTU13; OTU35; OTU51; OTU78 | undefined | OTU1; OTU28 |

**Table S4.** The common OTUs after supplying HR and HY to *H. axyridis*

| **Family** | **OTUs** | **Family** | **OTUs** |
| --- | --- | --- | --- |
| **Bacteria** |  |  |  |
| Enterobacteriaceae | OTU7; OTU1; OTU48 | Moraxellaceae | OTU5; OTU12;OTU46 |
| Staphylococcaceae | OTU2; OTU146;OTU9; | Streptococcaceae | OTU4; |
| Pseudomonadaceae | OTU3; OTU134 | Micrococcaceae | OTU14; OTU106; |
| Enterococcaceae | OTU13 | Sphingomonadaceae | OTU16 |
| Xanthomonadaceae | OTU29 | Bifidobacteriaceae | OTU24 |
| Burkholderiaceae | OTU31 | Corynebacteriaceae | OTU143 |
| Lachnospiraceae | OTU65 | Rhizobiaceae | OTU84 |
| undefined | OTU22 |  |  |
| **Fungi** |  |  |  |
| Wallemiaceae | OTU2 | Davidiellaceae | OTU7; OTU26;OTU20; |
| Pleosporaceae | OTU12 | Trichocomaceae | OTU13;OTU18;OTU35;OTU51; |
| undefined | OTU1; OTU41; OTU60 |  |  |

**Table S5.** The common OTUs after supplying HR and HM to *H. axyridis*

| **Family** | **OTUs** | **Family** | **OTUs** |
| --- | --- | --- | --- |
| **Bacteria** |  |  |  |
| Enterobacteriaceae | OTU7; OTU1 | Moraxellaceae | OTU12;OTU46 |
| Staphylococcaceae | OTU2; OTU146;OTU9; | mitochondria | OTU87 |
| Pseudomonadaceae | OTU134; OTU3 | Micrococcaceae | OTU14; |
| Enterococcaceae | OTU13 | Sphingomonadaceae | OTU16 |
| Comamonadaceae | OTU113 | Bifidobacteriaceae | OTU24 |
| Burkholderiaceae | OTU31 | Corynebacteriaceae | OTU143; OTU18 |
| Bradyrhizobiaceae | OTU116 | Weeksellaceae | OTU60 |
| undefined | OTU22 | Brevibacteriaceae | OTU28 |
| **Fungi** |  |  |  |
| Wallemiaceae | OTU2 | Chaetomiaceae | OTU44 |
| Davidiellaceae | OTU11;OTU26;OTU20; OTU6; OTU7 | Incertae_sedis | OTU54 |
| Trichocomaceae | OTU13;OTU35 OTU51; | Agaricostilbaceae | OTU79 |
| undefined | OTU1; | Nectriaceae | OTU85 |
